# A Method for Achieving Nanoscale Visual Positioning Measurement Based on Ultra-Precision Machining Microstructures

**DOI:** 10.3390/mi14071444

**Published:** 2023-07-19

**Authors:** Yihan Chen, Honglu Li, Zijian Zhu, Chenyang Zhao

**Affiliations:** School of Mechanical Engineering and Automation, Harbin Institute of Technology, Shenzhen 518055, China; 200310114@stu.hit.edu.cn (Y.C.); 21s153189@stu.hit.edu.cn (H.L.)

**Keywords:** precision measurement, image registration algorithm, microstructure

## Abstract

Microscopic visual measurement is one of the main methods used for precision measurements. The observation morphology and image registration algorithm used in the measurement directly affect the accuracy and speed of the measurement. This paper analyzes the influence of morphology on different image registration algorithms through the imaging process of surface morphology and finds that complex morphology has more features, which can improve the accuracy of image registration. Therefore, the surface microstructure of ultra-precision machining is an ideal observation object. In addition, by comparing and analyzing the measurement results of commonly used image registration algorithms, we adopt a method of using the high-speed SURF algorithm for rough measurement and then combining the robust template-matching algorithm with image interpolation for precise measurements. Finally, this method has a repeatability of approximately 54 nm when measuring a planar displacement of 25 μm.

## 1. Introduction

Precision positioning measurement is widely used in various precision instruments and CNC machine tools [1,2]. Currently, the commonly used precision positioning methods are divided into optical methods such as laser interference and optical encoding, and non-optical methods such as capacitance sensing and eddy current sensing [3,4]. These methods have certain limitations in range and measurement freedom, and the manufacturing of the equipment is complex and costly. Microscopic visual measurement, due to its advantages such as simple structure, ability to achieve multi-degree-of-freedom and large-range measurement, and wide application scenarios, has gradually become one of the main methods of precision measurement [5,6].

Microscopic visual measurement uses equipment such as cameras to obtain digital images of the observed object via microscopic imaging. Therefore, obtaining high-resolution observed objects is of great significance to microscopic visual measurement. The development of ultra-precision machining makes it possible to obtain high-resolution observed objects [7]. Ultra-precision machining (UPM) is an important mechanical material removal method, which is currently applied in many fields, such as optics, electronics, aerospace, and telecommunications [8]. Through ultra-precision machining, microstructured surfaces with nanometer surface roughness and sub-micrometer positional accuracy can be obtained, which can be used as observed objects for microscopic visual positioning measurement. The current mainstream methods mostly use periodic microstructures [9,10], but their machining accuracy directly affects the measurement accuracy, so the requirements for manufacturing and calibration are high, and the high similarity of periodic microstructures makes the image processing process complex.

In order to overcome the limitations of the positioning measurement technology based on periodic microstructures, our team proposed a microscopic visual measurement method based on non-periodic surface microstructures, using single-point diamond cutting technology to process non-periodic microstructures as observation objects [11,12]. The surface microstructure processed by this technology has richer changes in morphology compared to etching and carries more image information after microscopic imaging [13]. This method can process microstructure patterns of different geometric sizes according to different range requirements and eliminates tedious calibration operations, which can meet different occasions and usage requirements.

The non-periodic surface microstructure combined with image registration technology achieves microscopic measurement by registering and positioning the continuously obtained images of the microstructure after microscopic imaging. Commonly used image registration methods can usually be divided into three categories: feature-based registration such as SIFT, SURF, etc. [14]; gray information-based registration such as template matching [15]; and transformation domain-based registration such as phase correlation [16]. The three methods have certain differences in calculation speed, accuracy, and robustness, so they will show different effects when applied to microscopic measurements with high resolution and real-time small changes in imaging conditions.

In order to achieve higher precision and faster measurement, this paper proposes a method based on ultra-precision machining microstructures combined with SURF and template matching to achieve micro-displacement precision measurement. In the theoretical part of this paper, the optical imaging principle of the surface microstructure is analyzed in detail, the influence of surface morphology on imaging results is explored, and its influence on image registration is further analyzed. Afterwards, experiments were set up to compare the results of different image registration algorithms for processing ultra-precision machining microstructure and etching morphology, and we verify the influence of surface morphology on image registration, obtaining the conclusion of the superiority of ultra-precision machining microstructure for microscopic measurement. At the same time, by setting up experiments, we obtain the results of microscopic measurement based on ultra-precision machining microstructures using the three commonly used image algorithms and analyze the pros and cons of various algorithms. Finally, based on the conclusion of the previous step, a positioning method based on ultra-precision machining microstructures using SURF for rough measurement and template matching for precise measurement is proposed, and the results are analyzed.

## 2. Theory

### 2.1. Image Registration for Measuring Displacement

**Figure 1:**
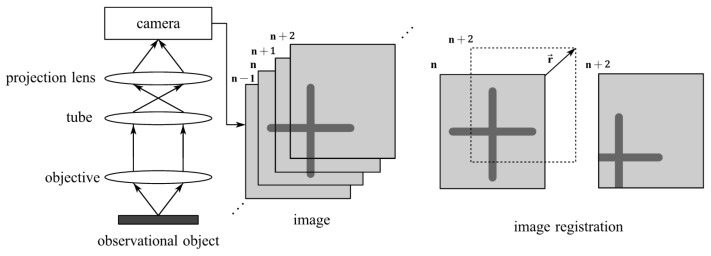
Steps of microscopic visual measurement. *n* represents the sequence number of the image and $\vec{r}$ represents the displacement of the object.

As shown in Figure 1, microscopic visual measurement is carried out in two steps. The first step is to microscopically image the observed moving object and obtain a continuous sequence of images. The second step is to register the images obtained from different positions, obtain the pixel displacement between the two images, and then calculate the actual displacement of the observed object using the conversion coefficient. Commonly used image registration methods can usually be divided into three categories. Therefore, this paper uses three different algorithms, namely, transformation domain-based phase correlation, gray information-based template matching, and feature-based SURF, to perform image registration.

#### 2.1.1. Phase Correlation

Phase correlation is a method of image registration based on spectral analysis. By performing Fourier transform on the images and analyzing the overlapping part of the frequency domain images, the transformation parameters between the two images are calculated. Let $\left[f_{1}\left(x,y\right)\right]$ be the pixel matrix of the original image, and when the image undergoes a translation transformation $\left(x_{0},y_{0}\right)$, its pixel matrix changes to $\left[f_{2}\left(x,y\right)\right]$. The transformation relationship between the two is as follows:

(1) $$f_{2}\left(x,y\right)=f_{1}\left(x-x_{0},y-y_{0}\right)$$

Taking the Fourier transform on both sides of the equation to the frequency domain $\left(u,v\right)$, we obtain the following:

(2) $$F_{2}\left(u,v\right)=e^{-2\pi j\left(ux_{0}+vy_{0}\right)}F_{1}\left(u,v\right)$$

Calculating the cross-power spectrum in the frequency domain, we obtain the following:

(3) $$e^{-2\pi j\left(ux_{0}+vy_{0}\right)}=\frac{F_{1}\left(u,v\right)\bar{F_{2}}\left(u,v\right)}{\left|F_{1}\left(u,v\right)\bar{F_{2}}\left(u,v\right)\right|}$$

Finally, performing an inverse Fourier transform on the cross-power spectrum yields an impulse function, which is almost zero at other positions except for the maximum value at $\left(x_{0},y_{0}\right)$. Therefore, by finding the peak position of the impulse function, we can calculate the translation parameters between the two images.

#### 2.1.2. Template Matching

**Figure 2:**
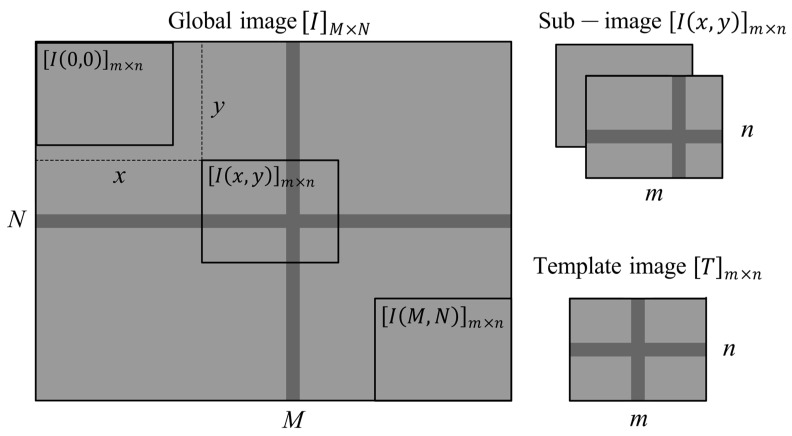
Template matching.

Template matching is a method of locating images based on gray information. As shown in Figure 2, let ${\left[T\right]}_{m\times n}$ be the gray matrix of the template image, ${\left[I\right]}_{M\times N}\;$ be the gray matrix of the global image, and ${\left[I\left(x,y\right)\right]}_{m\times n}$ be the sub-image cut out from the global image with the upper-left corner coordinates of $\left(x,y\right)$. The normalized cross-correlation coefficient (*NCC*) between the template image and the sub-image is defined as follows [17]:

(4) $${NCC}_{\left(x,y\right)}=\frac{\sum_{i=1}^{m}\sum_{j=1}^{n}\left[I\left(x+i,y+j\right)-\bar{I}\left(x,y\right)\right]\cdot \left[T\left(i,j\right)-\bar{T}\right]}{{\left[\sum_{i=1}^{m}\sum_{j=1}^{n}{\left[I\left(x+i,y+j\right)-\bar{I}\left(x,y\right)\right]}^{2}\right]}^{\frac{1}{2}}\cdot {\left[\sum_{i=1}^{m}{\sum_{j=1}^{n}\left(T\left(i,j\right)-\bar{T}\right)}^{2}\right]}^{\frac{1}{2}}}$$

where $\bar{T}$ is the average gray pixel intensity of the template image ${\left[T\right]}_{m\times n}$, and $\bar{I}\left(x,y\right)$ is the average gray pixel intensity of the sub-image ${\left[I\left(x,y\right)\right]}_{m\times n}$. The larger the value of *NCC*, the more similar the pixel gray distribution between the template image and the sub-image. When *NCC* is 1, the pixel gray distribution of the template image and the sub-image are exactly the same. Therefore, by finding the position with the maximum value of *NCC*, we can determine the relative displacement between the two.

#### 2.1.3. SURF

SURF (speeded up robust features) is a method of image registration based on feature matching with good invariance to geometric transformations, illumination, and other factors [18]. SURF uses the determinant value of the Hessian matrix as the feature point response detection. For any point $X=\left(x,y\right)$ in the image space with a scale of *σ*, the Hessian matrix is defined as follows:

(5) $$H\left(X,\sigma \right)=\left[\begin{array}{cc} L_{xx}\left(X,\sigma \right) & L_{xy}\left(X,\sigma \right)\\ L_{xy}\left(X,\sigma \right) & L_{yy}\left(X,\sigma \right) \end{array}\right]$$

where $L_{xx}\left(X,\sigma \right)$ represents the convolution of the Gaussian second-order partial derivative $\frac{\partial^{2}}{\partial x^{2}}$ with the image at X, and $L_{xy}\left(X,\sigma \right)$ and $L_{yy}\left(X,\sigma \right)$ are similar. To speed up the calculation, SURF uses a box filter to approximate the second-order partial derivatives of the Gaussian filter and replaces $L_{xx}$, $L_{xy}$, and $L_{yy}$ with $D_{xx}$, $D_{xy}$, and $D_{yy}$, respectively. The approximate determinant expression of the fast Hessian matrix can be obtained as follows:

(6) $$det\left(H\right)=D_{xx}D_{yy}-{\left(0.9D_{xy}\right)}^{2}$$

In order to extract the feature points, all the points in a 3 × 3 × 3 neighborhood are subjected to non-maximum suppression. Points with response values $det\left(H\right)$ greater than the neighboring 26 response values are selected as feature points, and then the image feature points are registered to achieve localization.

### 2.2. The Influence of Surface Microstructure

In order to explore the influence of surface microstructure defects on localization measurement, it is necessary to first analyze the imaging process of microstructure morphology. The imaging of surface microstructure is achieved via light reflection. For any reflection process, it can be described by the reflectance equation [19]:

(7) $$L_{0}=\int_{\Omega }f_{r}{\cdot L}_{i}\cdot (n\cdot \omega_{i})d\omega_{i}$$

where *n* is the normal vector, ω is the solid angle, $L_{0}$ and $L_{i}$ are the radiance of the outgoing and incoming light, and $f_{r}$ is a function related to the reflecting surface. Since surface microstructure is a typical non-optical plane, the light passes through the complex scattering process on the surface to form an image, and the reflection function adopts the bidirectional reflectance distribution function:

(8) $$L_{0}=\int_{\Omega }\left(k_{d}\frac{c}{\pi }+k_{s}\frac{DFG}{4\left(\omega_{0}\cdot n\right)\left(\omega_{i}\cdot n\right)}\right)L_{i}\left(p,\omega_{i}\right)n\cdot \omega_{i}d\omega_{i}$$

where $k_{d}$ and $k_{s}$ are the proportion coefficients of diffuse reflection and specular reflection, D is the normal distribution function, G is the geometric function, and F is the Fresnel equation.

D is related to the microplane normal unit vector h, macroplane normal unit vector n, and roughness α. The larger the α, the larger the function value of D. The expression of D is as follows:

(9) $$D\left(n,h,\alpha \right)=\frac{\alpha^{2}}{\pi {\left({\left(n\cdot h\right)}^{2}\left(\alpha^{2}-1\right)+1\right)}^{2}}$$

G represents the self-occlusion of the micro-surface, which causes light loss, and is related to the macroplane normal unit vector n, roughness α, and reflection direction unit vector v. The larger the roughness, the greater the occlusion. The expression of G is as follows:

(10) $$G\left(n,v,\alpha \right)=\frac{n\cdot v}{\left(n\cdot v\right)\left(1-\frac{{\left(\alpha +1\right)}^{2}}{8}\right)+\frac{{\left(\alpha +1\right)}^{2}}{8}}$$

F describes the ratio of the reflected light to the refracted light and is related to the microplane normal unit vector h, reflection direction unit vector v, and plane base reflectivity $F_{0}$. The expression of F is as follows:

(11) $$F\left(h,v,F_{0}\right)=F_{0}+\left(1-F_{0}\right){\left(1-\left(h\cdot v\right)\right)}^{5}$$

The value of $f_{r}$ for different surface structures is different. Therefore, under the same illumination conditions, the reflectivity of microstructures is different, which leads to different gray value distributions of the image after imaging. According to the reflectance equation, the normal distribution and roughness of the surface morphology determine its reflectivity. Microstructures with a high rate of change in normal distribution and roughness will also exhibit strong changes in pixel gray values, with a larger gradient in pixel gray values. When there is a sudden change in normal distribution and roughness, the pixels of the image at that location will also exhibit a sudden change, resulting in more obvious image features after imaging. The pixel distribution of the optical image is affected by surface morphology and processing precision. Therefore, the more complex the surface morphology is, the higher the accuracy and precision of image registration will be. The higher the processing precision is, the more controllable the precision of image registration will be.

Ultra-precision machining microstructure has a complex morphology, whereas the morphology of etched structures is relatively simple. Therefore, by taking ultra-precision machining microstructure and etched morphology as observation objects, respectively, the influence of surface morphology on image registration can be further compared and verified.

## 3. Experimental Setup

### 3.1. Microstructure Machining Experiment

**Figure 3:**
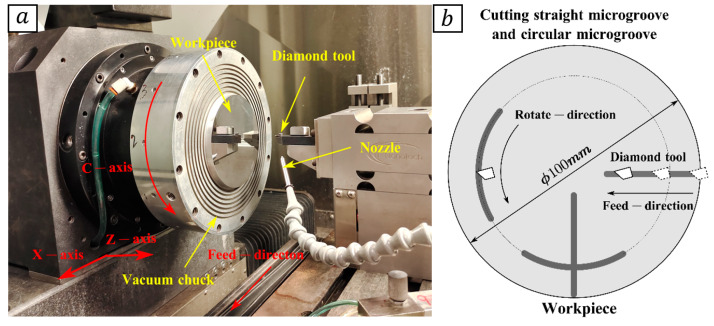
(**a**) Experimental setup; (**b**) workpiece.

The ultra-precision machining microstructure used in this paper was machined using a single-point diamond cutting method on a Moore ultra-precision lathe (Nanotech 450 UPL, Moore Nanotechnology Systems, Swanzey, NH, USA). The experimental setup and machining process are shown in Figure 3. During the SPDT (single-point diamond turning) process, the workpiece rotates along the C-axis, and the tool feeds along the *X*-axis direction. The rotational speed and feed rate are controlled to control the relative motion between the tool and the workpiece, thus realizing different machining morphologies. The machining is divided into three steps. In step 1, the workpiece surface is first turned to a mirror finish with a tool radius of 1 mm, a cutting depth of 5 μm, a spindle speed of 1500 r/min, and a feed rate of 0.002 mm/min. In step 2, circular micro-grooves are cut on the obtained mirror surface with a tool radius of 0.1 mm and a cutting depth of 3 μm. In step 3, linear micro-grooves are cut based on step 2, using a tool radius of 0.1 mm and a cutting depth of 3 μm.

**Figure 4:**
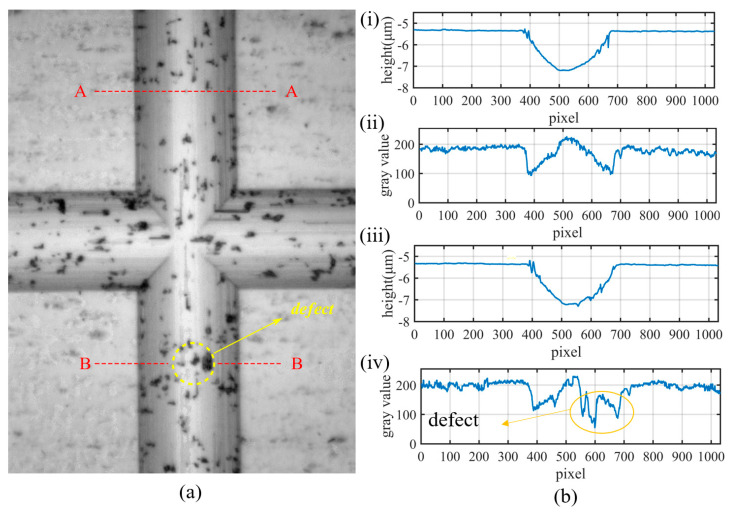
Machining results of ultra-precision machining microstructure; (**a**) grayscale image of intersecting grooves; (**b**) distribution of section morphology and grayscale of straight grooves; (i) morphology distribution of micro-groove A-A section; (ii) grayscale distribution of micro-groove A-A section; (iii) morphology distribution of micro-groove B-B section; (iv) grayscale distribution of micro-groove B-B section.

After the microstructure machining is completed, the machined surface is observed using an optical microscope, and a series of images are taken under the same lighting conditions. Figure 4 shows the results of microstructure processing. As shown in Figure 4a, there are small defects such as pits and scratches on the surface after machining. This is because there are unevenly distributed hard points inside the workpiece material. During the machining process, the hard points are exposed on the workpiece surface and fall off at the original position under the action of the tool, causing pits. They can also undergo tiny plastic deformation due to the action force of the tool, or follow the movement of the tool to produce scratches on the machined surface. The generated small defects have randomness in location and morphology, causing local mutations in the ultra-precision machining microstructure.

Figure 4b shows the depth and grayscale distribution of the ultra-precision machining microstructure at the A-A and B-B cross-sections. The processing quality of section A-A is good. The horizontal part of its depth distribution corresponds to the plane, and the arc part corresponds to the groove. The grayscale distribution exhibits a sudden change as the surface transitions from flat to grooved, whereas there is no significant mutation in the groove. On the other hand, the processing quality of the B-B cross-section is poor with three processing defects present. The depth distribution is roughly similar to that of the A-A cross-section, whereas the grayscale distribution exhibits three sudden changes in the groove, corresponding to the positions of the processing defects. This demonstrates that defects that only have small morphological mutations can have a significant impact on the grayscale after imaging.

### 3.2. Micro-Displacement Measurement Experiment

**Figure 5:**
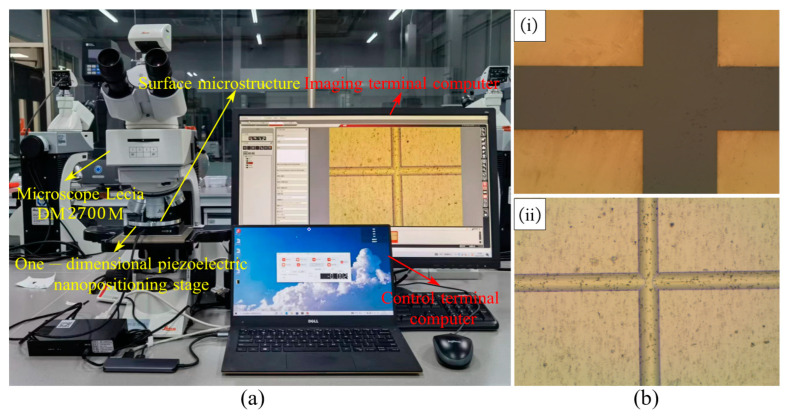
(**a**) Micro-displacement measurement system; (**b**) imaging results; (i) etched morphology; (ii) ultra-precision machining microstructure.

The micro-displacement measurement experimental system is composed of an optical microscopy imaging system, a micro-displacement control system, and the measured workpiece. The Leica DM2700 M microscope (Leica Microsystems GmbH, Wetzlar, Germany) was selected for the optical microscopy imaging system, and its imaging results were controlled and recorded by a computer at the imaging terminal. The P18.X single-axis piezoelectric micro-motion positioning stage from COREMORROW (Harbin, China) was selected for the micro-displacement control system, and the platform was driven by a computer at the control terminal to move the workpiece by several micrometers to the specified position, with a closed-loop resolution of 7 nm. The entire experimental system is shown in Figure 5a.

The objects selected for microscopic imaging are the etched morphology and the ultra-precision machining microstructure, with the observation position of the ultra-precision machining microstructure being the intersection of the straight groove and the annular groove on the workpiece surface, and the observation position of the etching being the intersection of two perpendicular grooves. The magnification was adjusted to 27.5×, and the imaging results showed a “cross” shape, as shown in Figure 5b.

**Figure 6:**
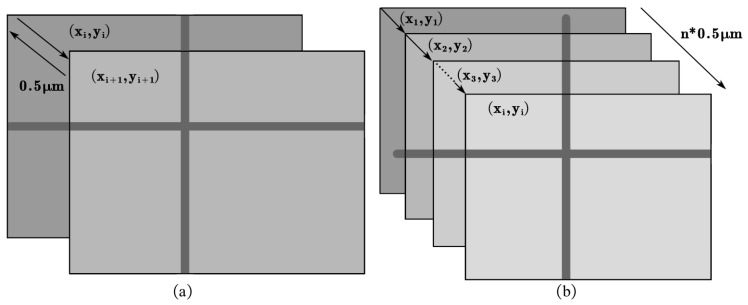
(**a**) Schematic diagram of reciprocal movement experiment; (**b**) schematic diagram of unidirectional movement experiment.

The micro-displacement measurement experiment was divided into two groups: the reciprocating movement experiment and the unidirectional movement experiment, as shown in Figure 6. In the reciprocating movement experiment, the initial position of the object was recorded as $\left(x_{1},y_{1}\right)$. The micro-displacement stage was controlled to move a small distance, and when it stabilized, the object’s position was recorded as $\left(x_{2},y_{2}\right)$. Then, the micro-displacement stage was controlled to move in the opposite direction to the initial position by the same distance, and the position of the object was recorded as $\left(x_{3},y_{3}\right)$. The experiment was repeated several times. After each movement, the object was optically imaged. In the unidirectional movement experiment, the initial position of the object was also recorded as $\left(x_{1},y_{1}\right)$. The micro-displacement stage was controlled to move the same distance in the same direction each time. After the i-th movement, the position of the object was recorded as $\left(x_{i+1},y_{i+1}\right)$. By performing image registration on the optical microscopy images, the pixel movement values, $a_{x}$ and $a_{y}$, in the *x* and *y* directions, respectively, were obtained. When calculating the distance between the measurement positions $\left(x_{i},y_{i}\right)$ and $\left(x_{j},y_{j}\right)$, the displacements ${∆}_{x}$ and ${∆}_{y}$ along the *x* and *y* directions, respectively, can be calculated using the following equations:

(12) $${∆}_{x}=\frac{a_{x}}{f_{x}},{∆}_{y}=\frac{a_{y}}{f_{y}}$$

where $f_{x}$ and $f_{y}$ are the conversion coefficients between pixels and actual sizes. The cumulative displacement recorded in each group of experiments ultimately presents a straight line passing through the origin. The slope of the line, which is the pixel shift value of adjacent two displacements, can be obtained using the least squares method. The conversion coefficient is obtained by dividing the actual displacement value input on the micro-displacement stage by the slope. The actual displacement Δ can be calculated using the Pythagorean theorem, as shown in the following equation:

(13) $$∆=\sqrt{{∆}_{x}^{2}+{∆}_{y}^{2}}$$

## 4. Results and Discussion

### 4.1. Different Surface Morphologies

**Figure 7:**
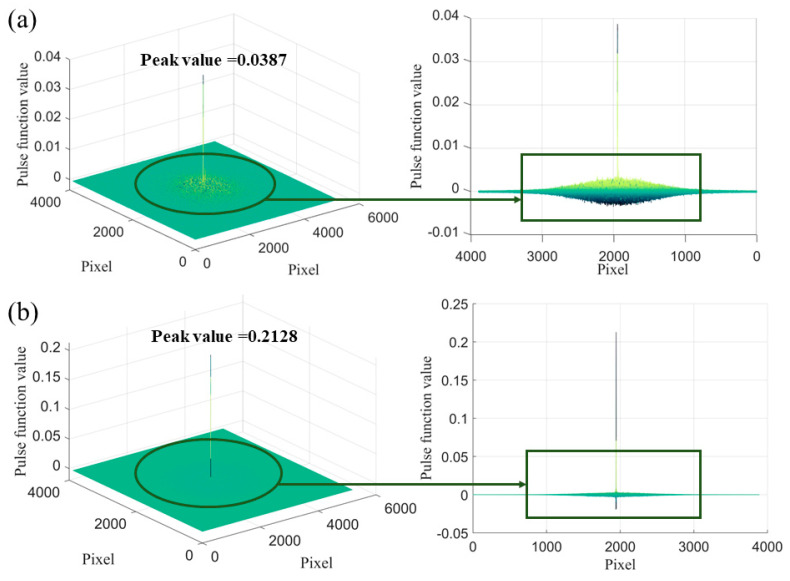
Three−dimensional pulse function of etched morphology and ultra−precision machining microstructure at each pixel position for calculating a displacement of 30 μm using phase correlation method. (**a**) Three−dimensional pulse function of etched morphology; (**b**) three−dimensional pulse function of ultra−precision machining microstructure.

Figure 7 shows the three-dimensional pulse function plots obtained by performing phase correlation calculations on the images captured before and after moving the etched morphology and ultra-precision machining microstructure by 30 μm, respectively. The *x*-axes and *y*-axes represent the pixel coordinates of the image, and the *z*-axis represents the pulse function value. The coordinates corresponding to the maximum value of the pulse function are the pixel values of the observed object’s translation. Figure 7a shows the three-dimensional pulse function plot of the etched morphology, and Figure 7b shows the three-dimensional pulse function plot of the ultra-precision machining microstructure.

As can be seen from the figures, the peak value of the pulse function for the etched morphology is less than 0.04, and there is significant fluctuation in a large range around the peak value. In contrast, the peak value of the pulse function for the ultra-precision machining microstructure is greater than 0.2, and the fluctuation in the non-peak region is small. This is because ultra-precision machining microstructures exhibit richer changes in pixel intensity and distribution after imaging, resulting in the ability to decompose more frequency and amplitude waves after Fourier transform. As a result, the spectrum of the microstructure becomes more complex. Therefore, after transformation into a pulse function, it has a sharp peak, which means that using the phase correlation method to measure the displacement of the ultra-precision machining microstructure has higher accuracy than that of the etched morphology.

**Figure 8:**
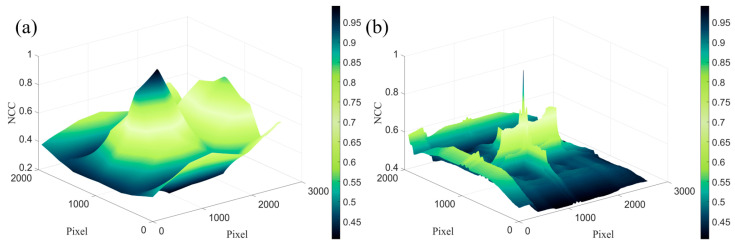
The distribution of *NCC* values in the global image of etched morphology and ultra-precision machining microstructure at each pixel position for calculating a displacement of 30 μm using the template-matching method. (**a**) The distribution of *NCC* values in the global image of etched morphology. (**b**) The distribution of *NCC* values in the global image of ultra-precision machining microstructure.

Figure 8 shows the distribution of the normalized cross-correlation (*NCC*) values in the global images obtained by template matching on the images captured before and after moving the etched morphology and ultra-precision machining microstructure by 30 μm, respectively. The image captured before moving is used as a global image, and sub-images from the moved image are captured as template images. Then, all positions in the global image are transversed to capture sub-images of the same size, matched with the template image, and the *NCC* value for each pixel position is calculated. The *x* and *y* axes represent the pixel coordinates of the image, and the *z*-axis represents the *NCC* value. A higher *NCC* value means that the template image and the matching image have a higher similarity. Figure 8a shows the distribution of the *NCC* values in the global image of the etched morphology, and Figure 8b shows the distribution of the *NCC* values in the global image of the ultra-precision machining microstructure.

As can be seen from the figures, the maximum *NCC* values of both the etched morphology and ultra-precision machining microstructure tend to reach 1, which means that both have high accuracy in measuring displacement using template matching. However, compared with the ultra-precision machining microstructure, the etched morphology has relatively high *NCC* values at nonoptimal matching points, whereas ultra-precision machining microstructures have smaller *NCC* values at nonoptimal matching points. This is because the etched morphology has a certain regional similarity, while the ultra-precision machining microstructure morphology has a large change, resulting in a larger change in grayscale distribution after imaging, which has a certain location uniqueness. Therefore, a larger *NCC* value of the ultra-precision machining microstructure is only obtained when the template image is coincident with the matching image. This makes the ultra-precision machining microstructure more robust in template matching. Due to the non-uniformity of the light intensity of the light source in both time and space, there may be slight differences in the lighting conditions before and after moving the camera. When the lighting conditions change, the image noise after imaging also changes, resulting in a change in the grayscale variance of the image. The *NCC* value at each position fluctuates to a certain extent. The sharp *NCC* peaks of ultra-precision machining microstructure images still have higher accuracy than the etching morphology when the lighting conditions change.

**Figure 9:**
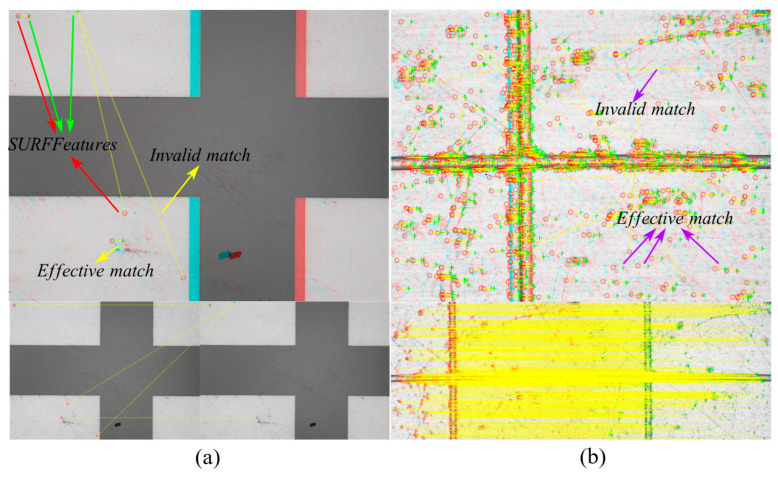
Feature point extraction of etched morphology and ultra-precision machining microstructure for calculating a displacement of 30 μm using the SURF algorithm. (**a**) Feature point matching result of etched morphology before displacement. (**b**) Feature point matching result of ultra-precision machining microstructure before and after displacement.

Figure 9 shows the feature-matching results obtained by using the SURF algorithm on the images captured before and after moving the etched morphology and ultra-precision machining microstructure by 30 μm, respectively. The extracted feature points and matching results are displayed, where the red circles represent the feature points in the image before translation, the green crosses represent the feature points in the image after translation, and the yellow lines represent the matching results. Figure 9a shows the matching result of the etched morphology, and Figure 8 and Figure 9 show the matching result of the ultra-precision machining microstructure.

As can be seen from the images, both effective and ineffective matches exist in the feature-matching process of the etched morphology and ultra-precision machining microstructure. This is because the imaging conditions of the observed object may slightly change before and after translation, resulting in noise in the imaging results and different feature points extracted before and after the translation. Therefore, the more feature points extracted, the more effective matches, and the higher the accuracy and precision of the measurement results. Due to the simple structure of the etched morphology, fewer SURF feature points can be extracted, whereas the complex morphology of the ultra-precision machining microstructure results in a larger number of extractable feature points and more effective matches. Therefore, when measuring displacement using the SURF feature matching method, the accuracy and precision of the ultra-precision machining microstructure are higher than those of the etched morphology.

During the SURF matching process of the ultra-precision machining microstructure, many small machining defects are extracted as feature points. This is because after imaging, the pixel grayscale gradient at the point of the small defect is large, and the determinant value of the Hessian matrix is large, making it a good feature point. However, due to the randomness of the distribution of machining defects, the matching property is poor, and ineffective matches may occur. Therefore, processing smaller and more regular morphologies to provide more features for matching is of great significance for our future research.

In summary, the ultra-precision machining microstructure has superior performance over the etched morphology in measuring displacement using image registration methods due to its richer morphology features, unique frequency spectrum distribution, and grayscale distribution. It has higher accuracy and precision, as well as stronger robustness. Therefore, the ultra-precision machining microstructure is superior to the etched morphology in displacement measurement using image registration methods.

### 4.2. Different Measurement Algorithms

**Figure 10:**
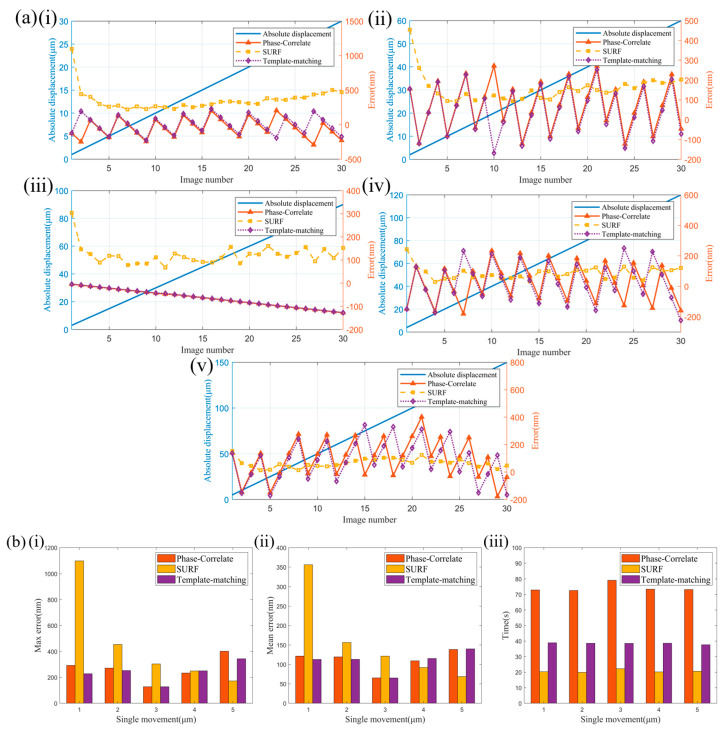
Results of calculating the absolute displacement of ultra−precision machining microstructure unidirectional movement using phase correlation method, template−matching method, and SURF feature extraction. (**a**) Measurement errors at different single displacements: (i) single displacement of 1 μm; (ii) single displacement of 2 μm; (iii) single displacement of 3 μm; (iv) single displacement of 4 μm; (v) single displacement of 5 μm. (**b**) Statistical results at different single displacements: (i) maximum measurement error; (ii) average measurement error; (iii) measurement time.

Figure 10 shows the results of calculating the absolute displacement of ultra-precision machining microstructure movement using phase correlation, template matching, and SURF matching methods. The ultra-precision machining microstructure was moved unidirectionally 30 times, and the displacement of each movement was the same for each experiment. In Figure 10a, the left *y*-axis represents the absolute displacement, the right *y*-axis represents the measurement error, and the *x*-axis represents the image sequence. The blue solid line represents the absolute displacement, the red triangle line represents the measurement error of the phase correlation method, the yellow square line represents the measurement error of the SURF algorithm, and the purple diamond line represents the measurement error of the template-matching method. Figure 10ai–v correspond to the displacement values of 1 μm, 2 μm, 3 μm, 4 μm, and 5 μm, respectively. In Figure 10b, the results of the three algorithms are compared. The red bar represents the phase correlation method, the yellow bar represents the SURF algorithm, and the purple bar represents the template-matching method. The *x*-axis represents the displacement value for each experiment. Figure 10bi represents the average measurement error for each experiment, Figure 10bii represents the maximum measurement error for each experiment, and Figure 10biii represents the time required for each experiment.

The results indicate that when the displacement value for each movement is 1 μm, the measurement accuracy of the SURF algorithm is lower, and the average and maximum measurement errors are much larger than the other two algorithms. As the displacement value for each movement increases, the measurement accuracy of the SURF algorithm gradually improves. When the displacement value for each movement reaches 5 μm, the measurement results of the SURF algorithm are significantly better than the other two algorithms due to the gradually decreasing error caused by incorrect matching with the increase in displacement value. The measurement errors of the phase correlation and template-matching methods are relatively stable and not strongly correlated with the displacement value for each movement.

In terms of measurement time, the size of the absolute displacement has little effect on the measurement time, and the SURF algorithm shows obvious superiority in measurement speed, followed by the template-matching algorithm, while the phase correlation method has the largest calculation amount and the longest measurement time.

When measuring larger displacements, the SURF algorithm shows obvious superiority over the other two algorithms, with higher measurement accuracy and faster measurement speed. However, when the displacement value for each movement is less than 4 micrometers, considering both measurement accuracy and measurement speed, the template-matching algorithm is the optimal choice.

### 4.3. Coarse-to-Fine Positioning Method

From Section 4.2, it can be seen that the SURF algorithm has high computational speed but unstable measurement accuracy, whereas the template-matching algorithm has a larger computational amount but robust and high accuracy under various conditions. Therefore, in this section, we propose a method that combines the advantages of both algorithms. Specifically, the SURF algorithm is used for coarse positioning of the image to narrow down the search range for template matching and reduce the computational amount, thus facilitating the subsequent fine positioning using template matching. In addition, to improve the measurement accuracy, bilinear interpolation is used to process the image.

**Figure 11:**
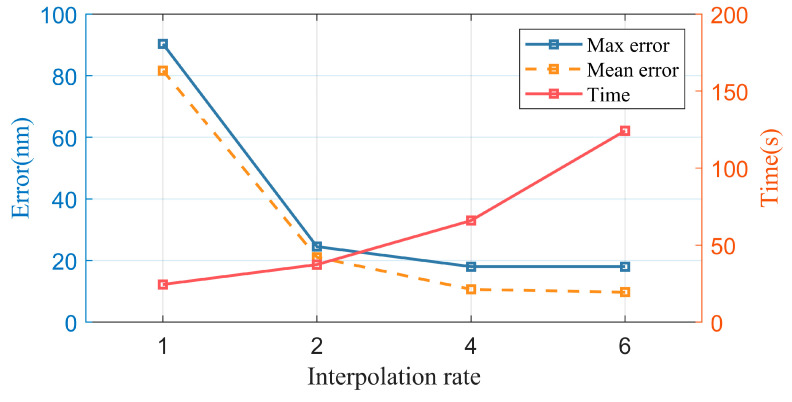
Results of measuring relative displacement using SURF coarse positioning and template-matching fine positioning.

Figure 11 shows the results of measuring the relative displacement using coarse positioning with SURF and fine positioning with template matching on ultra-precision machining microstructures that moved back and forth the same distance 30 times, with each movement distance of 25 μm. The absolute displacement between every two movements is measured and the difference between it and the actual displacement value input in the micro-displacement stage is calculated. The *x*-axis represents the interpolation factor for bilinear interpolation preprocessing of the image, with the experimental groups with no interpolation (1× interpolation), 2× interpolation, 4× interpolation, and 6× interpolation. The group without interpolation is used as a control for coarse measurement using SURF. The yellow line represents the average measurement error, the blue line represents the maximum measurement error, and the red line represents the measurement time.

The results show that both the average and maximum measurement errors decrease with increasing interpolation factor, and after 6× interpolation, the average and maximum measurement errors are less than 20 nm. When the interpolation factor is greater than 2×, the decrease in measurement error becomes less significant, which is due to overfitting caused by a too-high interpolation factor, leading to new errors. As the interpolation factor increases, the measurement time increases rapidly due to the sharp increase in computational amount, so it is not advisable to choose a large interpolation factor. When the interpolation factor is 4×, the measurement time is less than 70 s, the average measurement error is less than 15 nm, the maximum measurement error is less than 20 nm, and the repeatability is approximately 54 nm, which can achieve fast and high-precision measurement. 

## 5. Conclusions

This paper proposes a novel precision positioning method that combines an ultra-precision machining microstructure with SURF and template matching to achieve fast and high-precision microscopic visual measurement without calibration. The impact of different surface morphologies on imaging results is also analyzed, revealing that more complex surface morphologies lead to higher accuracy of image registration due to richer image information and features. Experimental verifications were conducted on ultra-precision machining microstructures with complex and simple morphologies, comparing three commonly used image registration algorithms in terms of the speed and accuracy of micro-displacement measurement. In addition, the proposed micro measurement method based on ultra-precision machining microstructure was experimentally tested, which combines SURF, template matching, and image interpolation to achieve fast and high-precision measurement. When the interpolation factor is four, the repeatability of the measurement is 54 nm when measuring a planar displacement of 25 μm.

This paper mainly considers the measurement of translation degrees of freedom without considering the influence of image scaling and rotation on the measurement results. Further research needs to consider the impact of scale transformation and increase the measurement of rotational degrees of freedom. In addition, the lighting conditions have a significant impact on the imaging process, thus directly affecting the measurement accuracy. However, this study did not explore the impact of lighting changes on measurement results. Therefore, further testing of the robustness of this method to lighting conditions is needed in subsequent research, and in-depth consideration of BRDF is needed to process more suitable microstructures via imaging mechanisms. Because the method proposed in this study is currently not suitable for large-scale measurements, its usage has been limited. In future research, we will propose a method to expand the range based on the current research results.

## Data Availability

Data sharing is not applicable to this article.
